# Multiscale Mechanisms and a Mechanism-Oriented Evaluation Framework for Warm-Mix Asphalt

**DOI:** 10.3390/ma19183839

**Published:** 2026-09-09

**Authors:** Xin Zhang, Ya Lu, Xinhai Liu

**Affiliations:** 1 School of Smart City Construction and Digital Twin, Chongqing Institute of Engineering, Chongqing 400056, China; 2 Guangdong Jiaoke Technology Development Co., Ltd., Guangzhou 510550, China; liuxinhai8721@sina.cn

**Keywords:** WMA, multiscale mechanisms, cross-scale evidence chain, mechanism-oriented evaluation framework

## Abstract

Warm-mix asphalt (WMA) reduces mixing and compaction temperatures through wax additives, chemical additives, and foaming techniques, but their dominant mechanisms, performance trade-offs, and evaluation priorities differ. This structured narrative review synthesizes evidence for Sasobit, Evotherm, and Advera as representative technologies across mixture, binder and mastic, interfacial and microstructural, and molecular scale. Sasobit-type wax additives improve construction-stage fluidity and high-temperature stability through viscosity–temperature regulation and wax crystallization, while low-temperature and fatigue risks require attention. Evotherm-type chemical additives enhance wetting and moisture resistance through surface-active adsorption, thin-film lubrication, and improved interfacial adhesion, with high-temperature shear resistance requiring verification. Advera-type zeolite foaming extends the compaction window through water release and microbubble formation, but residual moisture and wet-condition durability remain critical concerns. On this basis, technology-specific cross-scale evidence chains are established, and a mechanism-oriented evaluation framework is proposed, linking engineering scenarios, dominant mechanisms, reduced-temperature mix design feasibility, durability constraints, and applicability assessment. Mixture performance serves as the final criterion, while binder and interfacial evidence supports risk screening and molecular evidence provides mechanistic interpretation. The framework supports targeted material selection, experimental design, risk diagnosis, and process optimization.

## 1. Introduction

Against the backdrop of global climate change, the low-carbon transition of transportation infrastructure, and increasingly stringent health and safety requirements for road construction, the sustainability of asphalt pavement construction has received increasing attention. Energy consumption, greenhouse gas emissions, and asphalt fumes generated during asphalt mixture production, together with the continued consumption of non-renewable resources, have become major constraints on the sustainable development of road infrastructure [1,2,3,4,5]. Conventional hot-mix asphalt (HMA) is generally produced at approximately 150–180 °C, whereas Warm-mix asphalt (WMA) is typically produced at temperatures about 20–40 °C lower than those of equivalent HMA [6]. This reduction can decrease fuel consumption and construction-related fume emissions while also mitigating, to some extent, the short-term aging of the asphalt binder [1,2,3,7,8].

Existing studies indicate that WMA is not merely a temperature reduction technology but a coupled material–process system that simultaneously influences binder rheology, asphalt–aggregate interfacial adhesion, mixture air-void structure, and long-term durability [1,2,9,10,11,12]. Lower construction temperatures can reduce energy consumption and fume emissions while limiting the short-term aging of the asphalt binder [1,2,3,7,8,11,12]. However, lower production and compaction temperatures may also result in incomplete aggregate drying, inadequate compaction, discontinuous asphalt films, residual moisture at the asphalt–aggregate interface, reduced moisture resistance, and greater susceptibility to fatigue and low-temperature cracking [11,13,14,15]. Therefore, the engineering value of WMA should not be assessed solely on the basis of temperature reduction or changes in rotational viscosity. Instead, it should be evaluated comprehensively in terms of construction feasibility, pavement performance, and long-term durability risks [2,15,16,17].

Extensive research has been conducted on WMA in relation to mix design, the determination of mixing and compaction temperatures, pavement performance [2,11,17], incorporation of reclaimed asphalt pavement (RAP) contents [18,19,20], interfacial moisture damage [13,14,15,21,22], and molecular simulation [23,24], providing a substantial foundation for its engineering application. Nevertheless, two major limitations remain in the existing body of review and experimental research. First, the roles and evidential boundaries of different analytical scales have not been clearly defined. Improvements in mixture compaction, reductions in binder viscosity, interfacial wetting, and molecular adsorption are often discussed without clearly distinguishing their respective scales or functions. Consequently, the pathways through which material-scale mechanisms translate into engineering performance remain insufficiently understood. Second, a differentiated evaluation rationale for distinct WMA technologies has yet to be fully established. Many studies continue to evaluate WMA using individual indicators, such as the magnitude of temperature reduction, rotational viscosity, rutting resistance, or the tensile strength ratio (TSR) [13,15,17,21]. Relatively few studies have systematically determined, on the basis of dominant mechanisms, which indicators are appropriate for each technology, how potential risks should be identified, and how engineering applicability should be assessed. Nevertheless, existing reviews and technical guidelines provide a foundation for developing such a differentiated evaluation framework.

To address these limitations, this review selects Sasobit, Evotherm, and Advera as representative wax additive-based, chemical additive-based, and foaming-based WMA technologies, respectively, and systematically examines their mechanisms at the mixture, binder, interface and microstructure, and molecular scales. Building on this multiscale synthesis, a mechanism-oriented framework for evaluating WMA technologies is developed to support targeted material selection, experimental design, risk identification, and process optimization.

The principal objectives of this review are to: (1) summarize the technical classifications and applicability characteristics of wax additive-based, chemical additive-based, and foaming-based WMA technologies; (2) analyze the mechanisms of action and potential risk sources of the three technology categories at the mixture, binder, interface and microstructure, and molecular scales; (3) construct cross-scale evidence chains that clarify the dominant action targets and evaluation priorities of each technology category; and (4) propose a mechanism-oriented evaluation framework and illustrate its application logic for material selection and risk assessment through a hypothetical engineering scenario. Overall, this review seeks to advance WMA evaluation from a temperature reduction-centered approach towards an integrated framework that combines mechanisms, performance indicators, risk identification, and engineering scenarios.

## 2. Review Methodology and Evidence Synthesis

### 2.1. Review Methodology

This study adopted a structured narrative review approach to synthesize the multiscale mechanisms and engineering evaluation of warm-mix asphalt (WMA) technologies. The literature search was conducted primarily using Google Scholar and covered publications from 2000 to 2026. The literature search was completed in June 2026, and additional studies identified during manuscript preparation were evaluated using the same criteria. The search strategy combined general WMA-related terms (“warm mix asphalt” OR “WMA” OR “warm-mix asphalt”) with technology-specific and mechanism-related terms, including “wax additive”, “Sasobit”, “Fischer–Tropsch wax”, “chemical additive”, “Evotherm”, “surfactant”, “adhesion promoter”, “foaming”, “zeolite”, “Advera”, “water release”, “rheology”, “viscosity”, “surface free energy”, “adhesion”, “moisture damage”, “RAP”, “molecular dynamics”, and “multiscale mechanism”.

The literature selection consisted of title and abstract screening followed by full-text assessment. Studies were included when they provided evidence related to WMA mechanisms or engineering evaluation at the mixture, binder and mastic, interfacial and microstructural, or molecular scales. Studies were excluded if they lacked sufficient information on materials or experimental methods, were unrelated to WMA mechanisms or engineering performance, or substantially duplicated previously published studies. Peer-reviewed journal articles constituted the primary evidence base, supplemented by technical reports, field studies, and engineering guidelines where they provided relevant mechanistic or engineering evidence.

During evidence synthesis and manuscript preparation, additional studies were identified through reference-list screening, citation tracking, and targeted supplementary searches addressing specific mechanisms and performance issues. These studies were evaluated using the same inclusion and exclusion criteria and incorporated when they provided relevant evidence for cross-scale mechanism interpretation.

**Figure 1:**
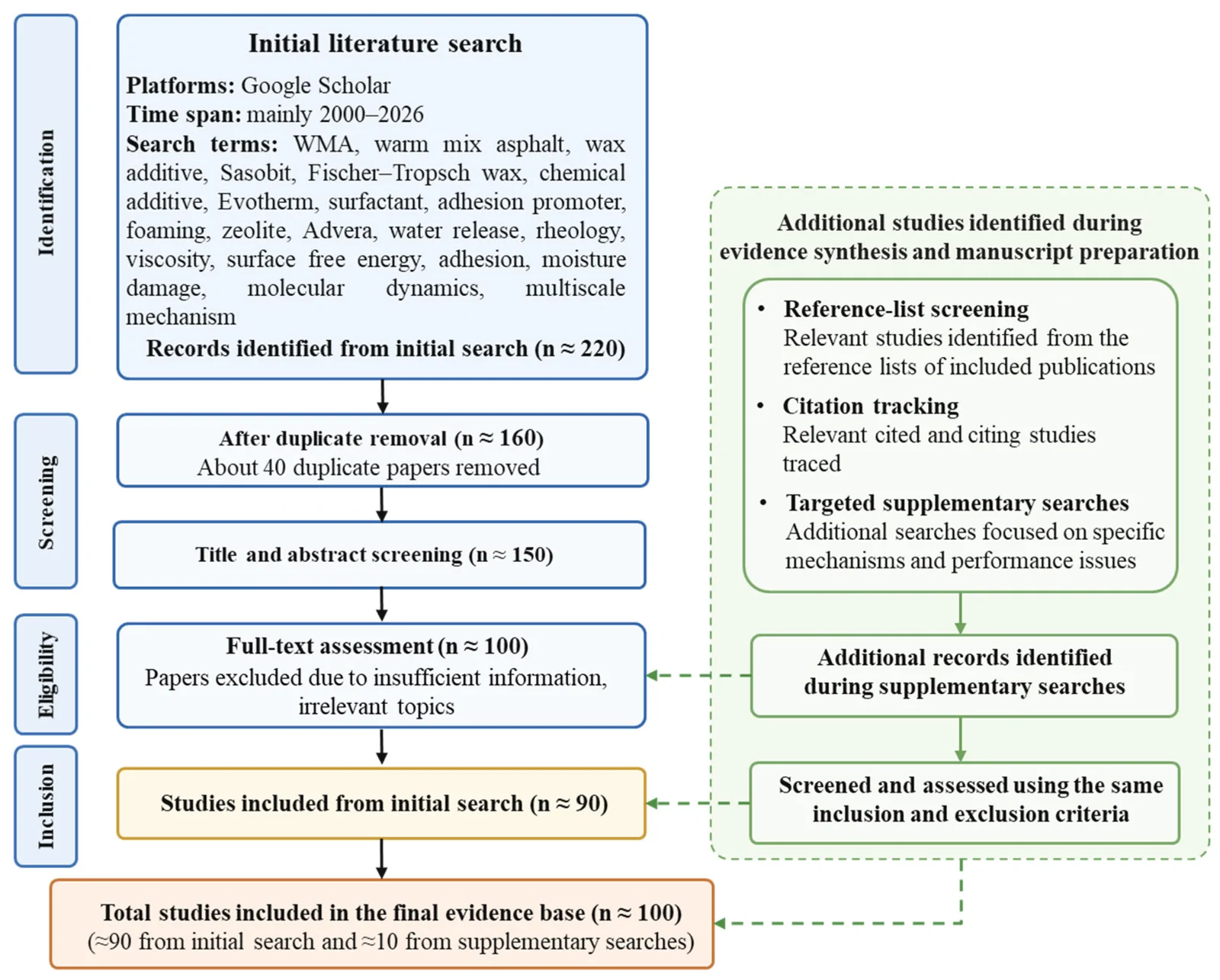
Literature identification and screening process.

Approximately 100 publications were ultimately included in the evidence base, with most studies published between 2005 and 2026. Because of substantial heterogeneity in materials, additive types, testing conditions, and evaluation methods, quantitative meta-analysis was not performed. Instead, qualitative evidence synthesis was conducted to distinguish consistent trends, condition-dependent findings, and contradictory results reported across different studies. The selected studies were organized according to WMA technology category and analytical scale to support the construction of cross-scale mechanism chains and the mechanism-oriented evaluation framework proposed in this study. The literature identification, screening, and supplementary search procedures are summarized in Figure 1.

### 2.2. Evidence Classification

The selected literature was organized using a two-dimensional classification framework based on WMA technology category and analytical scale. According to the technology category, the reviewed studies were classified into three groups: wax additives, chemical additives, and foaming techniques.

Sasobit, Evotherm, and Advera were selected as representative cases because relatively comprehensive cross-scale evidence is available for these products across multiple analytical scales. Findings from these products were treated primarily as product-level evidence and were extended to broader WMA categories only when comparable mechanisms or trends were supported by additional studies.

According to the analytical scale, the available evidence was classified into four levels: (1) mixture scale, covering compactability, air-void structure, rutting resistance, moisture resistance, fatigue performance, and low-temperature cracking resistance; (2) binder and mastic scale, covering viscosity–temperature behavior, rheological responses, phase transition characteristics, aging behavior, and flow characteristics; (3) interfacial and microstructural scale, covering asphalt–aggregate wetting, interfacial adhesion, surface free energy, moisture-induced debonding, and microstructural damage; (4) molecular scale, covering diffusion, adsorption, crystallization, intermolecular interactions, molecular rearrangement, and moisture migration. Table 1 presents the resulting three-category × four-scale evidence matrix. The matrix summarizes the distribution of available evidence and provides a basis for constructing cross-scale mechanism chains; however, it does not quantitatively weight evidence strength. Support for cross-scale mechanism chains was assessed qualitatively according to cross-scale consistency, methodological complementarity, and engineering relevance, as described in Section 2.3.

### 2.3. Criteria for Establishing Cross-Scale Evidence Chains

Cross-scale evidence chains were established qualitatively based on three considerations: cross-scale consistency, methodological complementarity, and engineering relevance. These criteria evaluate whether observations across different scales support a consistent mechanistic interpretation, whether different analytical approaches provide complementary evidence, and whether lower-scale observations can plausibly explain larger-scale performance responses.

No fixed minimum number of studies was required for each analytical scale because evidence availability varies among WMA technologies and analytical levels. Mechanism chains supported by multiple studies and complementary cross-scale evidence were considered to have stronger support, whereas condition-dependent or contradictory findings were retained to identify potential limitations and applicability boundaries.

## 3. Classification of WMA

According to the 2024 EAPA technical briefing, common WMA production technologies can be broadly classified into organic additives, chemical additives, and foaming technologies [25]. Previous reviews have indicated that this mechanism-based classification provides a more rational basis for interpreting differences among WMA technologies in terms of binder viscosity, asphalt–aggregate interfacial adhesion, moisture susceptibility, low-temperature cracking resistance, rutting resistance, RAP compatibility, and life-cycle assessment results [1,2,9,11,12,17,18,25,26,27,28].

### 3.1. Wax Additives

Organic WMA additives are generally composed of wax-based organic materials [25,29,30]. Typical materials include Fischer–Tropsch synthetic waxes, Montan waxes, fatty acid amides, bio-waxes, and other organic viscosity-reducing agents [26,29,30]. These additives are mainly used to regulate the temperature sensitivity of asphalt binders, allowing mixtures to maintain sufficient workability at lower mixing and compaction temperatures [26,29,30,31,32,33,34]. Representative products include Sasobit, Asphaltan-B, FT wax, Montan wax, and several bio-wax products [26,29,30]. Existing studies have suggested that wax WMA additives can achieve a temperature reduction of approximately 20–30 °C; however, their effectiveness is jointly affected by asphalt source, additive melting point, dosage, mixture type, and construction temperature regime [1,2,11,17,26,29,30,31,32,33,34].

From an engineering perspective, the advantages of organic WMA additives lie in their relatively direct temperature reduction effect. Their application scenarios include conventional dense-graded mixtures, SMA, high-temperature heavy-duty pavement sections, and selected high-RAP systems [11,15,17,18,19,20,26,27,28,33]. However, organic WMA additives should not be regarded simply as “temperature-reducing agents”. Their low-temperature toughness, fatigue performance, compatibility with polymer-modified asphalt, such as SBS-modified binders, and phase-structure stability after long-term aging still need to be verified through performance-based tests.

### 3.2. Chemical Additives

Chemical WMA additives are usually composed of surface-active compounds, adhesion promoters, emulsifying agents, or anti-stripping agents [35,36,37,38,39,40]. Compared with organic WMA additives, chemical additives do not necessarily produce a significant reduction in the bulk viscosity of the binder [35,36,37]. Instead, they mainly expand the mixing and compaction temperature window by improving wetting at the asphalt–aggregate interface, aggregate coating, and inter-particle lubrication [35,36,37]. Representative products include Evotherm, Rediset, Cecabase, and Zycotherm [26,39,40]. Relevant studies have indicated that the typical temperature reduction range of chemical WMA additives is approximately 20–40 °C [1,2,11,17,26,34,37,39,40]. However, because the compositions of different products vary considerably, their applicability should be comprehensively evaluated by considering aggregate mineralogy, asphalt source, RAP content, and moisture-stability requirements.

The reported engineering benefits of chemical WMA additives mainly include improved aggregate coating at lower mixing temperatures and enhanced compactability, while improvements in moisture resistance are more dependent on the specific asphalt–aggregate system and conditioning conditions [35,36,37,38,39,40]. Because their mechanism is not equivalent to bulk viscosity reduction alone, construction temperatures should not be determined solely using the equiviscous-temperature method.

### 3.3. Foaming Techniques

Foamed WMA technology introduces a small amount of water into hot asphalt, where the water vaporizes and forms asphalt foam [26,41,42]. This process improves binder dispersion, aggregate coating, and compaction performance within a limited time window [41,42,43,44,45]. According to the method of water introduction, foaming technologies can be divided into direct water-injection foaming and indirect foaming using water-bearing minerals [42]. The former relies on nozzles, pumping systems, and asphalt plant control systems to inject a controlled amount of water into hot asphalt, whereas the latter uses minerals containing crystalline water, such as zeolites, to release water at elevated temperatures and generate controlled foaming [42,43,44,45]. Representative technologies include mechanical water-injection foaming, WAM-Foam, Aspha-min, and Advera [42].

The engineering advantages of foaming technology include lower demand for external chemical additives, a high degree of equipment-based control, and suitability for large-scale production [27,28,42]. It also has strong synergistic potential for warm recycling of RAP [27,28]. Its application effectiveness is closely related to foaming water content, asphalt temperature, foam expansion ratio, half-life, aggregate moisture content, RAP moisture content, mixing sequence, and construction time window [41,43,44,45]. Compared with organic and chemical additives, foaming technology is more dependent on process control. Therefore, from the perspective of technological classification, it should be regarded as a WMA route governed by the combined effects of materials, equipment, and process parameters.

### 3.4. Comparative Classification of the Three WMA Technologies

Table 2 summarizes the classification basis, representative materials, typical temperature reduction ranges, and engineering application scenarios of the three major WMA technology categories.

## 4. Multiscale Mechanisms of WMA

This section examines WMA mechanisms across four scales: the mixture scale, the binder and mastic scale, the interfacial and microstructural scale, and the molecular scale. Sasobit, Evotherm, and Advera are selected as representative wax additives, chemical additives, and zeolite-based foaming techniques, respectively, to construct technology-specific cross-scale evidence chains for the three WMA categories.

Sasobit, Evotherm, and Advera are used in this review as representative cases rather than as universal proxies for all technologies within their respective categories. Sasobit primarily represents Fischer–Tropsch wax additives and may not fully capture the behavior of Montan waxes, fatty acid amides, or bio-based waxes. Evotherm products may differ in formulation, carrier system, and product generation, whereas Advera represents water-releasing zeolite-based foaming rather than mechanical water-injection foaming. Accordingly, findings obtained for these products are treated as direct product-level evidence. Any extension of these findings to broader WMA categories is regarded as a conditional category-level inference and requires sufficient similarity in dominant mechanism, material composition, and application conditions. In particular, test methods and interpretive criteria based on foam expansion ratio and half-life, which are commonly used for mechanically foamed asphalt, should not be transferred directly to all zeolite-based WMA systems without method-specific validation.

### 4.1. Mixture-Scale Mechanisms

The macroscopic mixture scale serves as the primary level for engineering verification of WMA mechanisms. At this scale, indicators such as aggregate coating ratio, Superpave gyratory compactor (SGC) compaction curves, air-void content (Va), mixture density, Marshall stability, indirect tensile strength (ITS), TSR, Hamburg wheel-tracking test (HWTT) and Asphalt Pavement Analyser (APA) rutting performance, flow number, dynamic modulus, fatigue life, and resistance to low-temperature cracking are used to determine whether mixtures retain adequate constructability and in-service reliability under reduced-temperature conditions [46,47,48]. Existing mix design and field-performance studies indicate that the ability of a mixture to achieve the target compaction level at the intended temperature reduction is a fundamental criterion for evaluating WMA applicability [49,50]. Moreover, WMA performance should not be assessed solely on the basis of temperature reduction magnitude or changes in rotational viscosity; degree of compaction, moisture resistance, and long-term performance should be considered together [49,50].

For Sasobit-type wax additives, the mixture-scale response is primarily characterized by improved compactability and enhanced rutting resistance [51]. The NCAT report by Hurley and Prowell showed that Sasobit improved compactability under both gyratory and vibratory compaction conditions. In mixtures containing PG 64-22 binder, the average air-void content was reduced by up to approximately 0.87%, and rutting potential was generally reduced. However, production at lower temperatures could increase moisture susceptibility [51]. Using dynamic modulus, TSR, four-point bending fatigue, flow number, and APA rutting tests, You et al. further demonstrated that the mixture-scale response to Sasobit depends jointly on dosage, mixing temperature, and mixture structure [52].

Additional studies indicate that Sasobit generally improves high-temperature stability [29,32,33,53]. However, variations in RAP content, compaction at reduced production temperatures, or aging state may make fatigue performance and moisture resistance limiting factors [33,52]. Therefore, at the mixture scale, the effects of Sasobit should not be summarized solely as “rutting reduction.” A more appropriate evidence chain is “improved compactability–enhanced rutting resistance–potential constraints on cracking and moisture resistance.”

The principal mixture-scale responses reported for Evotherm-type chemical additives include retained compactability at reduced production temperatures, while improvements in moisture resistance are more dependent on the specific material system and conditioning conditions [41,44,54,55]. NCAT Report 06-02 and subsequent field studies indicate that Evotherm can maintain adequate compaction at construction temperatures below those used for HMA [44,48,54]. In some systems, visible stripping was reduced, moisture resistance was improved, and TSR values were comparable to or higher than those of control mixtures [41,44,54,55]. Because chemical additives do not necessarily increase the bulk stiffness of the binder, their engineering benefits are often more clearly reflected in aggregate coating ratio, degree of compaction, TSR, HWTT, moisture-conditioned strength, and field density [33,41,44,54,55].

The mixture-scale effect of Advera-type zeolite foaming additives is primarily associated with extending the effective short-term compaction window [56]. Previous studies and engineering assessments have shown that Advera can improve mixture compactability and facilitate construction at reduced production temperatures through internally stored water release and transient microfoaming effects [28,42,44,52]. However, mixture performance indicators, including TSR, wet APA or HWTT, dynamic modulus, and fatigue response, may vary depending on material characteristics and testing conditions [52,56]. These findings indicate that the effectiveness of zeolite-based foaming techniques depends not only on whether foam is generated but also on foam half-life, the time available for compaction, and adequate control of residual moisture and moisture susceptibility.

### 4.2. Binder-Scale Mechanisms

The binder and mastic scale represents the material response level connecting mixture-scale performance with interfacial mechanisms. It addresses whether a warm-mix additive alters viscosity at mixing temperatures, viscoelastic behavior under service conditions, the shear resistance of thin mastic films, phase transition behavior, short-term aging, and susceptibility to long-term aging [32,33,37,57,58,59,60,61,62]. Common methods include rotational viscosity (RV), dynamic shear rheometry (DSR), multiple stress creep and recovery (MSCR), frequency sweeps, bending beam rheometry (BBR), linear amplitude sweep (LAS) testing, rolling thin-film oven and pressure aging vessel (PAV) conditioning, differential scanning calorimetry (DSC), Fourier-transform infrared spectroscopy (FTIR), saturates, aromatics, resins, and asphaltenes (SARA) analysis, gel permeation chromatography (GPC), and mastic lubrication or friction testing [32,33,37,57,58,59,60,61,62].

At the binder scale, the dominant mechanism of Sasobit is regulation of the bulk viscosity–temperature relationship [32,33,57,58,60,61]. Studies by Arega and Bhasin, among others, indicate that, unlike many chemical additives, Sasobit can partly compensate for the lower early-life stiffness associated with reduced short-term aging at lower production temperatures. Its action can be interpreted as viscosity reduction caused by wax melting at elevated temperatures, followed by stiffness enhancement through crystallization during cooling [60]. Saed et al. investigated Sasobit-modified warm recycled binders and found that Sasobit improved workability and high-temperature rutting resistance but reduced low-temperature flexibility [59]. These findings indicate that low-temperature relaxation capacity and fatigue cracking resistance are key constraints for this type of material. Therefore, Sasobit should be evaluated using a combination of RV, MSCR, BBR, LAS, DSC, FTIR, and SARA analysis rather than rotational viscosity alone.

The binder-scale response of Evotherm differs from that of Sasobit. Its dominant mechanism is not wax-crystal-induced stiffening but the reduction in thin-film shear resistance, improvement of lubrication, and regulation of rheological balance by surface-active components [37,58,63]. Studies of Evotherm-DAT show that it can improve the workability of rubberized asphalt and reduce construction temperatures, although high-temperature performance may be slightly lower than that of the corresponding system without a warm-mix additive [64,65,66]. When surfactants are combined with foaming, foam half-life may also be extended; however, excessive dosage may reduce $G^{*}/\sin\delta$ and compromise high-temperature stability [67]. Therefore, binder-scale evaluation of Evotherm should be combined with interfacial tests.

Advera exhibits a transient and process-dependent response at the binder and mastic scale [68]. Comparisons between hydrated and dehydrated Advera indicate that hydrated Advera produces a water release-induced foaming effect together with a particle-filling effect, whereas dehydrated Advera primarily reflects the filling effect of zeolite particles. Because zeolite is insoluble in asphalt, the presence of solid particles and gradual water release may complicate the interpretation of conventional steady-state viscosity and DSR results [42,60,68]. Advera should therefore be regarded as a coupled material–process system involving the superposition of transient microfoaming and residual mineral-particle effects. Its evaluation should therefore emphasize water release behavior, comparisons with dehydrated Advera, residual moisture measurements, and rheological responses, while foam expansion, half-life, and foam index may also be considered where applicable to the specific zeolite system.

### 4.3. Interfacial and Microstructural-Scale Mechanisms

The interfacial and microstructural scale focuses on contact, spreading, adsorption, and adhesion between asphalt films and aggregate mineral surfaces, as well as stripping behavior after moisture intrusion [39,69,70,71,72,73,74,75]. Common methods include contact angle measurement, surface free energy analysis, work of adhesion, work of debonding, energy ratio, binder bond strength (BBS) pull-off testing, atomic force microscopy (AFM) adhesion force measurement, scanning electron microscopy and fluorescence microscopy, the Hamburg stripping inflection point, and observation of failure morphology after freeze–thaw conditioning [70,71,72,73,74,75,76,77,78]. Unlike the binder scale, the interfacial scale emphasizes the specific asphalt–aggregate combination rather than the bulk properties of the binder alone [69,71,72,73,74,75,76,77,78].

Interfacial evidence for Sasobit indicates that its effects on spreading and surface free energy characteristics are aggregate-dependent [39,69,71,72,76,79,80,81]. Wasiuddin et al. found that Sasobit reduced the contact angle and increased the total surface free energy of the binder, although changes in energy ratio differed between Davis limestone and Snyder granite [79]. Ghabchi et al. also reported that Sasobit and Advera could reduce moisture damage potential in some asphalt–aggregate combinations but did not perform satisfactorily in strongly acidic granite systems [76]. In addition, aging pathways such as ultraviolet exposure and RTFO/PAV conditioning may further alter the interfacial adhesion behavior of Sasobit [39,75,80,81].

The interfacial mechanism of Evotherm is supported by more direct evidence. Ghabchi et al. measured surface free energy using the Wilhelmy plate method and a universal sorption device and found that 0.75% Evotherm significantly increased the work of adhesion in asphalt–limestone and asphalt–granite systems while reducing the work of debonding in the presence of water [76]. Together with the review by Button et al., FHWA technical information, and subsequent studies of surface free energy and moisture-conditioned interfacial behavior, these findings support an Evotherm mechanism chain of “surface-active adsorption–enhanced wetting–reduced work of moisture-induced debonding.” However, its effectiveness remains influenced by product generation, binder source, and aggregate mineralogy [26,82,83,84].

The interfacial mechanism of Advera is dual in nature. When zeolite releases water and forms microbubbles, the effective volume and mobility of the asphalt phase temporarily increase, allowing the asphalt film to spread more readily over aggregate surfaces. However, after foam collapse, residual water that is not removed or is retained within aggregate pores may form weak interfacial water films and reduce adhesion under wet conditions [26,27,42,60,68,74,78]. In recent years, the combination of AFM and molecular dynamics has provided a new evidence pathway for interfacial mechanisms by linking nanoscale adhesion force, molecular-scale work of adhesion, and mixture-scale moisture damage [75,85,86].

### 4.4. Molecular-Scale Mechanisms

Molecular-scale analysis is used to elucidate the fundamental origins of phenomena observed at the mixture, binder and mastic, and interfacial scales. At this scale, molecular dynamics (MD) simulations are commonly combined with AFM–MD cross-scale validation, SARA analysis and surface free energy (SFE) measurements [73,75,87,88]. These approaches are used to characterize molecular and physicochemical descriptors such as mean square displacement (MSD), diffusion coefficient, radial distribution function (RDF), fractional free volume (FFV), cohesive energy density (CED), glass transition temperature ($T_{g}$), adsorption energy, interaction energy, work of adhesion, shear stress, and molecular orientation [74,75,87,88,89,90,91,92,93,94,95].

The molecular mechanism of Sasobit-type wax additives involves the coupled regulation of molecular mobility, component distribution, cohesive state, and interfacial interactions after long-chain alkane or Fischer–Tropsch wax molecules enter the multicomponent SARA system of asphalt [96,97,98,99,100]. At elevated temperatures, wax molecules remain dispersed within the asphalt continuous phase, potentially increasing local free volume and promoting the mobility and diffusion of lighter fractions [96,97,98,99,100]. During cooling, wax chains become increasingly oriented and form localized crystalline domains, thereby restricting the mobility of asphaltene–resin structures and increasing cohesive energy density and system rigidity [96,97,98]. In high-RAP systems, wax molecules may facilitate initial contact between virgin and aged binders, although crystallization-induced constraints may limit subsequent interdiffusion [92,99]. Therefore, diffusion coefficient, free volume distribution, molecular orientation, wax crystallization behavior, and asphaltene aggregation state should be considered together.

Because publicly available atomistic models representing the proprietary Evotherm formulation are limited, molecular-scale discussion should focus on Evotherm-type surface-active chemical additives rather than on a product-specific molecular structure. MD shear and friction models show that surface-active molecules can reduce shear resistance at the asphalt–mineral interface through polar-head adsorption and nonpolar-chain lubrication [90,100]. However, molecular-scale evidence also indicates that lubrication does not necessarily correspond to enhanced adhesion under all conditions. Mineral surface characteristics, moisture, asphalt component models, and force-field selection can all influence the calculated work of adhesion and interfacial interaction energy [75,89,101].

The molecular mechanism of Advera-type zeolite foaming additives should be interpreted through three coupled processes: crystalline water release, molecular rearrangement within the asphalt phase, and competitive adsorption by residual water [95,102]. When bound water is released from zeolite pores during heating, microbubbles form and temporarily disturb the asphalt continuous phase, altering local density, free volume, and molecular mobility. After foam collapse, residual water may migrate toward the asphalt–aggregate interface and compete with polar asphalt components for active adsorption sites on mineral surfaces, thereby reducing the work of adhesion and increasing susceptibility to stripping [89,95,102].

Molecular-scale indicators should not be treated as engineering acceptance limits but as tools for mechanistic interpretation and early risk screening. For Sasobit, priority descriptors include MSD, diffusion coefficient, $T_{g}$, CED, FFV, and wax crystallization or phase transition behavior [23,93,94,98,99,100,101]. For Evotherm, adsorption energy, shear stress, RDF, molecular orientation, interfacial interaction energy, and competitive water adsorption should be emphasized [73,89,90,94,100,101]. For Advera, water diffusion pathways, local density distribution, cohesive interactions, work of adhesion, and work of debonding should be considered [26,74,77,78,89,95,101,102]. These indicators should be cross-validated against upper-scale tests such as RV, DSR/MSCR, SFE, BBS, TSR, HWTT, and freeze–thaw damage tests. The scale-specific evaluation priorities and principal risks associated with the three representative WMA technologies are summarized in Table 3.

### 4.5. Cross-Scale Integration of Mechanism–Performance Pathways

The three WMA technology categories exhibit distinct cross-scale mechanism–performance pathways. For Sasobit-type wax additives, the dominant mechanism chain can be summarized as “wax dispersion and crystallization–viscosity–temperature regulation–interfacial modification–improved compactability and rutting resistance, accompanied by potential low-temperature cracking and fatigue risks.” For Evotherm-type chemical additives, the dominant mechanism chain is “surface-active adsorption–thin-film lubrication–enhanced wetting and adhesion–maintained compactability and moisture resistance under reduced production temperatures.” For Advera-type zeolite foaming, the dominant mechanism chain is “crystalline water release–microbubble formation–temporary improvement in workability and aggregate coating–residual moisture-induced interfacial risk.” The scale-specific manifestations and corresponding evaluation priorities of these mechanism chains are summarized in Table 4.

Overall, evidence supporting reduced-temperature workability mechanisms is relatively consistent, whereas moisture resistance, cracking-related performance, aging response, and long-term durability remain strongly dependent on material characteristics and testing conditions.

## 5. Mechanism-Oriented WMA Evaluation Framework

Building on the multiscale mechanism analysis presented above, this review proposes a mechanism-oriented framework for evaluating WMA technologies. The framework translates engineering scenarios, dominant mechanisms, cross-scale evidence, and identified risks into an operational pathway for material selection, experimental design, and process optimization. The dominant mechanisms, performance responses, and evaluation priorities identified in Section 4 provide the scientific basis for the engineering evaluation framework developed in this section. Section 5 therefore focuses on integrating these cross-scale findings into indicator selection, performance verification, engineering applicability assessment, and feedback-based adjustment.

### 5.1. Basic Logic of the Evaluation Framework

The central principle of the framework is to select technology-specific combinations of indicators according to the dominant action targets of different WMA technologies and to translate the resulting evidence into engineering applicability decisions. Its logic follows the sequence “scenario definition–dominant mechanism identification–indicator selection–performance verification–risk mitigation and feedback adjustment.”

Mixture-scale performance serves as the final engineering decision criterion; binder-scale evidence provides process constraints and identifies rheological or material response risks; interfacial and microstructural evidence supports risk screening, particularly for adhesion and moisture-related damage; and molecular-scale evidence provides mechanistic interpretation and early risk identification.

From an engineering perspective, mixture-scale performance remains the final basis for applicability decisions. When material systems are complex, environmental conditions are sensitive, or test results are inconsistent, binder- and mastic-scale, interfacial, and molecular evidence can be used to trace the origins of performance variation and potential failure, thereby guiding targeted material and process optimization. The functional roles of different scales in mechanism-oriented WMA evaluation are summarized in Table 5.

### 5.2. Evaluation Procedure

Mechanism-oriented WMA evaluation can be implemented through a six-stage procedure: scenario definition, preliminary material screening, mix design verification, durability assessment, applicability classification, and feedback-based adjustment. This procedure is compatible with established WMA mix design methods and field-performance verification practices. Before evaluation, the engineering scenario and design constraints should be defined, including the target temperature reduction, traffic loading level, climatic conditions, aggregate mineralogy, RAP content and moisture, construction season, mixing equipment, and compaction conditions. The applicable specifications and project-specific performance requirements should also be identified at this stage, and the corresponding acceptance thresholds should be defined before testing rather than prescribed universally by the present framework.

During preliminary material screening, indicators should be selected according to the WMA technology category and its dominant action target. For wax additives, priority should be given to the viscosity–temperature relationship, DSC phase transition behavior, dynamic shear rheometer and MSCR responses, and low-temperature rheological risks. For chemical additives, priority indicators include aggregate coating ratio, contact angle, SFE, work of adhesion, and work of moisture-induced debonding. For zeolite-based foaming techniques, priority indicators include water release behavior, residual moisture, and foam collapse behavior, while expansion ratio, foam half-life, and foam index may be considered where applicable to the specific system. If preliminary screening indicates that the target temperature reduction is unattainable or that pronounced low-temperature embrittlement, moisture-induced debonding, or residual moisture risks are present, the system should be revised before proceeding to full mixture design verification.

Under the target reduced-temperature conditions, mix design verification should examine SGC compaction curves, air-void content, density, VMA, VFA, asphalt film continuity, and aggregate coating quality. These indicators determine whether the mixture can achieve the target compaction level and satisfy the volumetric design requirements. Durability assessment should then be conducted using technology-specific indicators. For wax additives, rutting resistance, low-temperature cracking resistance, and fatigue performance should be evaluated together. For chemical additives, TSR, HWTT, BBS, moisture-conditioned adhesion, and freeze–thaw damage should be prioritized. For zeolite-based foaming techniques, TSR, moisture-conditioned APA or HWTT, freeze–thaw strength retention, residual moisture, and foam collapse behavior should be evaluated.

**Figure 2:**
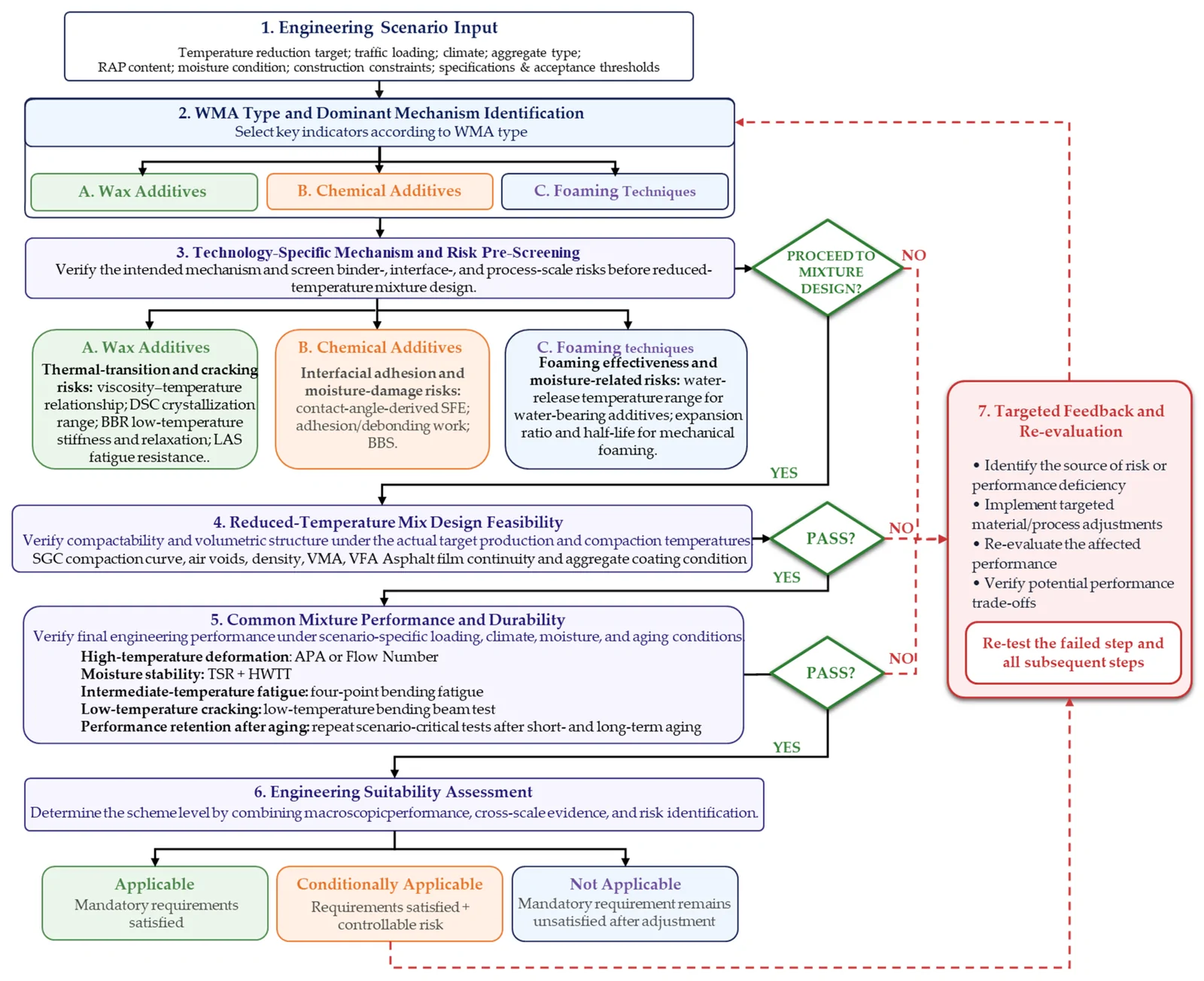
Mechanism-oriented framework for evaluating WMA technologies. Note: Solid arrows indicate the primary evaluation pathway, while dashed arrows indicate feedback and iterative adjustment within the framework.

**Figure 3:**
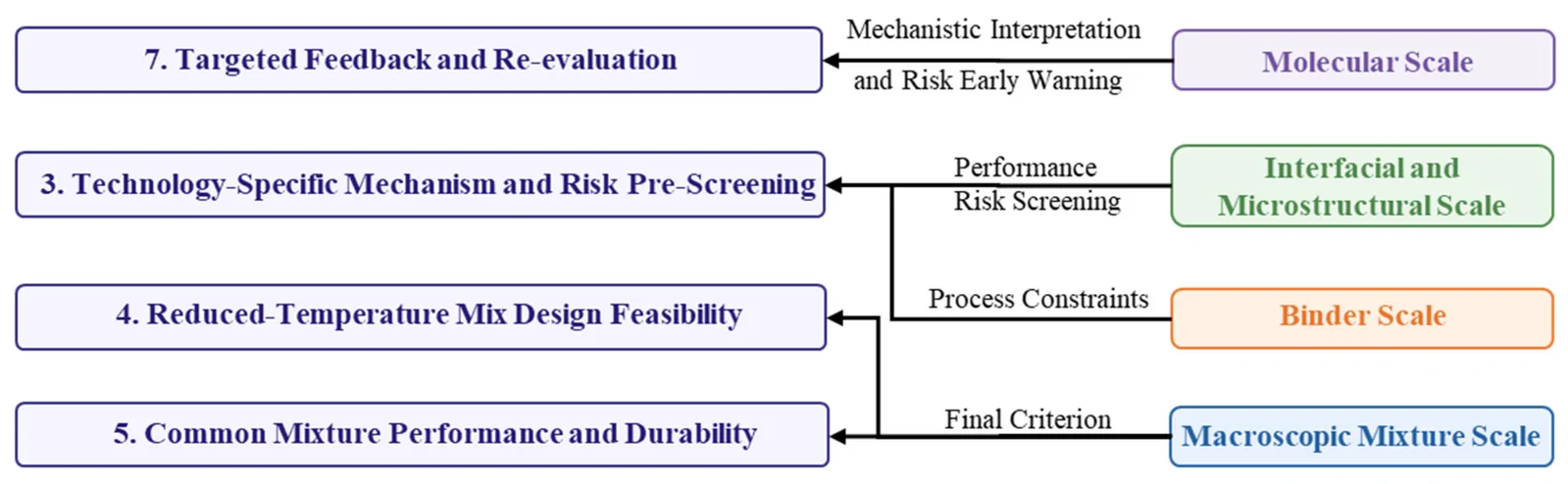
Cross-scale evidence chain.

On the basis of the evaluation results and the predefined acceptance criteria, a WMA scheme can be classified as applicable, conditionally applicable, or not applicable. A scheme may be classified as applicable when all mandatory mixture-scale performance requirements are satisfied under the target reduced-temperature conditions and the supporting lower-scale evaluations do not identify unresolved engineering risks requiring further mitigation or verification. A scheme should be classified as conditionally applicable when the mandatory mixture-scale performance requirements are satisfied, but one or more identifiable and controllable engineering risks remain and require targeted mitigation or further verification. A risk is considered controllable when its likely source can be identified, a feasible material or process adjustment can be implemented, and the affected performance can be reassessed after modification. A scheme should be classified as not applicable when one or more mandatory mixture-scale performance requirements cannot be satisfied under the target reduced-temperature conditions, including after reasonable material or process adjustment and targeted reassessment. The proposed mechanism-oriented framework for evaluating WMA technologies is illustrated in Figure 2. The cross-scale evidence chain supporting mechanism interpretation and indicator selection is presented in Figure 3.

### 5.3. Illustrative Scenario-Based Application

To illustrate the application of the proposed framework, a hypothetical heavy-traffic pavement project in a hot and humid region of southern China is considered. The project uses an asphalt mixture containing 30% RAP and requires the mixing and compaction temperatures to be reduced by approximately 30 °C relative to those used for the corresponding HMA control. The region is characterized by high summer pavement temperatures and frequent rainfall, while the selected aggregate exhibits moderate susceptibility to moisture-induced damage. In addition, the relatively high RAP content may increase effective binder stiffness and susceptibility to intermediate- and low-temperature cracking. The evaluation must therefore address compactability at the target mixing and compaction temperatures, resistance to permanent deformation, moisture durability, fatigue resistance, low-temperature cracking resistance, and performance retention after aging. An HMA mixture prepared with the same binder, aggregate, gradation, and RAP content should be used as the reference. Acceptance criteria for each evaluation should be defined in advance on the basis of project specifications and non-inferiority requirements relative to the HMA control.

During technology identification, wax additives, chemical additives, and foaming techniques should all be considered candidate solutions; however, none should be regarded as inherently superior solely on the basis of its technology category. For wax additives, binder melting–crystallization behavior and potential losses in low-temperature relaxation and fatigue resistance should be screened. For chemical additives, priority should be given to asphalt–aggregate wetting, interfacial adhesion, and resistance to moisture-induced debonding. For foaming techniques, the water release profile or the foam expansion and collapse characteristics should be evaluated according to the specific foaming mechanism. Following technology-specific pre-screening, all candidate mixtures should be evaluated at the target mixing and compaction temperatures using Superpave gyratory compaction, volumetric properties, aggregate coating quality, and asphalt film continuity. For foamed mixtures, residual moisture and the effective foaming and compaction window should also be assessed. Only candidates that achieve acceptable volumetric structure, aggregate coating, and compactability should proceed to the common mixture performance evaluation gate.

To illustrate how the framework can support mechanism-oriented diagnosis, consider a scenario in which all three candidate mixtures satisfy the compactability and volumetric requirements in Step 4, but one candidate exhibits a relatively low TSR or an early stripping response in the HWTT during Step 5. A conventional, technology-agnostic evaluation would identify only inadequate moisture durability, whereas the proposed framework uses evidence from the preceding steps to diagnose the underlying source of failure. For a chemical additive, a concurrent reduction in moisture-conditioned BBS would indicate inadequate interfacial adhesion, suggesting that the additive dosage, anti-stripping treatment, or aggregate moisture content should be adjusted. For a foaming technique, excessive residual moisture or a mismatch between foam stability and the mixing–compaction sequence would instead indicate the need to adjust the water dosage or release profile, foaming temperature, mixing and compaction timing, and curing conditions. For a wax additive, aggregate coating quality, air-void structure, and asphalt film continuity under reduced-temperature conditions should be examined, together with the potential effects of wax crystallization and binder stiffening. After corrective action, the failed evaluation gate and any downstream gates affected by the modification should be reassessed. Under this hot–humid, high-RAP scenario, the three WMA routes therefore lead to different screening priorities and corrective actions. The framework does not presume that any technology category is inherently superior; rather, suitability is determined by whether scenario-specific risks can be controlled while the common mixture performance requirements are satisfied. Accordingly, the same mixture-scale failure may lead to different material- or process-specific adjustments depending on the underlying mechanism. This hypothetical case is intended to illustrate the decision logic of the framework and does not constitute experimental or field validation. The evaluation results and applicability classification of the hypothetical WMA scheme are summarized in Table 6.

### 5.4. Limitations and Future Research

Several limitations of the present review and the proposed framework should be acknowledged.

First, the available WMA literature exhibits substantial heterogeneity in binder source, aggregate mineralogy, additive dosage, RAP content, aging condition, production temperature, conditioning protocols, and testing methodologies. These differences limit direct quantitative comparisons among studies and may partly explain the inconsistent performance outcomes reported in the literature. Therefore, quantitative meta-analysis was not conducted; instead, this review focused on the consistency, variability, and applicability boundaries of mechanism–performance relationships reported across studies.

Second, Sasobit, Evotherm, and Advera were selected as representative cases because relatively comprehensive multiscale evidence is available for these products. However, these products should not be assumed to represent all wax additives, chemical additives, or foaming techniques. Accordingly, product-specific observations were extended to broader technology categories only when similar mechanisms or performance trends were supported by additional studies involving materials with comparable functional characteristics. In addition, the quantity and maturity of available evidence are unevenly distributed among WMA technology categories and analytical scales. Mixture- and binder-scale studies are generally more abundant than interfacial or molecular-scale investigations; therefore, some cross-scale mechanism chains are supported by stronger evidence than others.

Third, the practical decision thresholds within the proposed framework cannot be universally fixed. The classification of a WMA technology as applicable, conditionally applicable, or not applicable should ultimately be determined according to local specifications, climatic conditions, traffic conditions, material characteristics, and project-specific performance requirements.

Finally, the proposed mechanism-oriented evaluation framework requires further validation through dedicated experimental programs and field projects. The scenario presented in Section 5.3 is intended to illustrate the application logic of the framework rather than serve as formal validation. Future research should therefore validate the framework using independent laboratory datasets, field trials, and long-term pavement performance data while further refining technology-specific evaluation indicators and decision thresholds under diverse engineering conditions.

## 6. Conclusions

In the context of sustainable asphalt pavement construction, WMA evaluation should consider its mechanisms, performance responses, and associated risks. Using a structured narrative review, this study synthesizes multiscale evidence and develops a mechanism-oriented evaluation framework. The main conclusions are as follows:

1. The three representative WMA technologies exhibit distinct mechanism–performance pathways. Wax additives are mainly associated with temperature-dependent rheological modification and phase transition, chemical additives with interfacial interactions, and foaming techniques with transient water-induced workability improvement. These differences result in technology-specific benefits and risks. Evidence from Sasobit, Evotherm, and Advera should be generalized only when comparable mechanisms and material characteristics are supported by additional studies.
2. The mechanism-oriented framework shown in Figure 2 links engineering scenario definition, dominant mechanism identification, targeted screening, reduced-temperature mixture design and performance assessment, and applicability classification. It provides a structured basis for material selection, experimental design, risk diagnosis, and process adjustment according to technology-specific mechanisms and project conditions.
3. Evidence across different analytical scales serves distinct functional roles rather than having equivalent engineering significance. As summarized in Table 5 and Figure 3, molecular-, binder-, and interfacial-scale evidence primarily supports mechanistic interpretation, process constraints, and risk identification, whereas mixture-scale performance remains the final engineering criterion. Cross-scale consistency strengthens interpretation but cannot replace direct mixture performance verification.
4. The framework remains limited by study heterogeneity, uneven evidence distribution, the limited transferability of product-specific evidence, and the absence of independent experimental or field validation. Future research should validate and refine the framework using laboratory, field, and long-term pavement performance data under different climatic, traffic, RAP, and material conditions.

## Figures and Tables

**Table 1:** Evidence distribution of representative WMA technologies across analytical scales.

WMA Technology	Mixture	Binder/Mastic	Interface/Microstructure	Molecular
Wax Additives	6	7	9	6
Chemical Additives	6	7	5	5
Foaming Techniques	5	3	7	3

Note: A single study may provide evidence across multiple analytical scales; therefore, the reported values should not be interpreted as additive counts of independent studies. The table summarizes the distribution of available evidence across representative WMA technologies and analytical scales and is intended to illustrate evidence coverage rather than quantitatively rank evidence strength.

**Table 2:** Classification, representative materials, and applicability of three WMA technologies.

Technology Type	Classification Basis and Representative Materials	Typical Temperature Reduction Range	Engineering Application Scenarios
Wax Additives	Wax-based organic materials; Sasobit, Asphaltan-B, FT wax, Montan wax, bio-wax, etc.	Approximately 20–30 °C; affected by melting point, dosage and asphalt composition.	Conventional dense-graded mixtures, SMA, high-temperature heavy-duty pavement sections and some high-RAP systems; low-temperature and fatigue risks should be verified.
Chemical Additives	Surfactants, adhesion promoters and emulsifying components; Evotherm, Rediset, Cecabase, Zycotherm, etc.	Approximately 20–40 °C; affected by aggregate mineralogy, asphalt source and moisture condition.	Systems requiring improved coating at lower mixing temperatures, enhanced compactability, acidic aggregates, high RAP contents or higher resistance to moisture damage.
Foaming Techniques	Direct water-injection foaming or water release foaming using water-bearing minerals; mechanical foaming, WAM-Foam, Aspha-min, Advera, etc.	Approximately 20–40 °C; affected by foaming water content, asphalt temperature and equipment parameters.	Large-scale production in asphalt plants, warm recycling of RAP and projects requiring fewer external additives.

**Table 3:** Scale-specific evaluation priorities and principal risks for the three WMA technologies.

WMA Technology	Mixture-Scale Priorities	Binder and Mastic Priorities	Interfacial and Molecular Priorities	Principal Risks
Wax Additives	SGC compaction, Va, APA/HWTT, flow number, dynamic modulus	RV, DSR/MSCR, DSC, BBR, LAS, SARA analysis	MSD, diffusion coefficient, $T_{g}$, CED, work of adhesion, contact angle/SFE	Reduced low-temperature relaxation capacity; fatigue cracking; incomplete blending in RAP systems; aggregate-dependent adhesion
Chemical Additives	Aggregate coating ratio, degree of compaction, TSR, HWTT, moisture-conditioned strength	RV, DSR/MSCR, FTIR, SARA analysis, mastic friction/lubrication	Adsorption energy, shear stress, molecular orientation, SFE, BBS, work of adhesion/debonding	Sensitivity to product formulation or generation and aggregate mineralogy; insufficient high-temperature shear resistance under excessive lubrication
Foaming Techniques	SGC compaction, Va, TSR, wet APA/HWTT, dynamic modulus	Expansion ratio, foam half-life, foam index, residual moisture, hydrated/dehydrated comparison	Water diffusion pathways, local density distribution, work of adhesion/debonding, moisture-conditioned interfacial observations	Short effective foaming window; residual moisture; interfacial water film formation and stripping; inadequate durability under wet conditions

Note: The listed risks represent potential mechanism-related concerns rather than universal outcomes; their occurrence and severity depend on material composition, additive dosage, environmental exposure, and production or conditioning conditions.

**Table 4:** Mechanism chains for the three WMA technologies.

WMA Technology	Molecular Starting Point	Binder and Mastic Response	Interfacial Behavior	Mixture-Scale Outcome	Mechanism-Derived Evaluation Priorities
Wax Additives	Dispersion, diffusion, and cooling-induced crystallization of long-chain wax molecules	Reduced viscosity during construction and increased stiffness under service conditions	Changes in spreading and work of adhesion, depending on aggregate mineralogy and moisture	Improved compactability and rutting resistance	Balance among construction-stage fluidity, high-temperature stability, and low-temperature/fatigue resistance
Chemical Additives	Oriented adsorption of surface-active molecules	Thin-film lubrication and reduced mixing resistance	Enhanced wetting, increased work of adhesion, and reduced work of moisture-induced debonding	Maintained coating and compaction at reduced production temperatures, with improved moisture resistance	Combined evaluation of wetting, adhesion, resistance to stripping, and high-temperature shear resistance
Foaming Techniques	Crystalline water release and microbubble formation	Foam expansion, half-life, and foam index define the effective construction window	Temporary coating improvement, while residual moisture may weaken the interface	Extended compaction window with possible variations in moisture-conditioned performance	Integrated evaluation of the effective foaming window, residual moisture, and durability under wet conditions

Note: The mechanism chains summarize dominant mechanism–performance tendencies rather than universally fixed outcomes; individual responses may vary with material and testing conditions. Evidence for Sasobit, Evotherm, and Advera is primarily product-specific and should be generalized only to systems with comparable formulation, dominant mechanism, and engineering conditions.

**Table 5:** Functional roles of different scales in mechanism-oriented WMA evaluation.

Scale	Primary Evaluation Objects	Functional Role	Engineering Significance
Macroscopic mixture scale	Compactability, air-void structure, rutting resistance, moisture resistance, fatigue performance, and low-temperature cracking resistance	Final engineering decision criterion	Determines whether the WMA scheme satisfies specification requirements under the target reduced-temperature conditions
Binder and mastic scale	Viscosity–temperature relationship, rheology, phase transition behavior, aging, low-temperature relaxation, and fatigue response	Process constraint	Determines whether bulk material responses support mixture-scale performance and identifies rheological trade-offs
Interfacial and microstructural scale	Wetting, adhesion, moisture-induced debonding, moisture intrusion, and interfacial damage	Risk screening	Assesses whether moisture damage and interfacial-failure risks are controllable
Molecular scale	Diffusion, adsorption, crystallization, cohesive and interaction energies, and moisture migration	Mechanistic interpretation and early risk identification	Explains performance differences, traces the origins of failure, and identifies potential failure mechanisms

**Table 6:** Scenario-specific application of the proposed mechanism-oriented WMA evaluation framework.

WMA Technology	Step 3: Technology-Specific Pre-Screening	Step 4: Reduced-Temperature Mix Design Gate	Step 5: Emphasis of the Common Performance Gate	Dominant Risks and Targeted Feedback
Wax Additives	Viscosity–temperature relationship and DSC crystallization range; BBR and LAS when required by the engineering scenario	SGC compaction curve, air-voids, density, VMA, VFA, and aggregate coating quality	APA or flow number; four-point bending fatigue; low-temperature bending test on asphalt mixtures; retention of critical performance after aging	If cracking risk is excessive, adjust the wax type or dosage, base-binder grade, rejuvenator dosage, or compaction temperature.
Chemical Additives	Contact angle-derived SFE, work of adhesion and debonding, and dry/wet BBS	SGC compactability, volumetric properties, aggregate coating, and asphalt film continuity	TSR and HWTT, together with the remaining common high-temperature, cracking, and aging-related performance requirements	If wet-state adhesion is inadequate, adjust the additive dosage, anti-stripping treatment, aggregate moisture, or mixing sequence.
Foaming Techniques	Water release temperature range for water-bearing additives; expansion ratio and half-life for mechanical foaming	SGC compactability, volumetric properties, effective workability window, residual moisture, and aggregate coating	TSR and HWTT, together with the remaining common high-temperature, cracking, and aging-related performance requirements	If residual moisture or wet-state durability is inadequate, adjust the release profile, foaming temperature, mixing–compaction timing, or curing conditions.

## Data Availability

No new data were created or analyzed in this study. Data sharing is not applicable to this article.
